# (6*S*,7*S*,8*S*,8a*S*)-6-Ethyl-3-oxo-1,2,3,5,6,7,8,8a-octa­hydro­indolizine-7,8-diyl diacetate

**DOI:** 10.1107/S1600536812005144

**Published:** 2012-02-10

**Authors:** Viktor Vrábel, Ľubomír Švorc, Peter Šafář, Štefan Marchalín

**Affiliations:** a Institute of Analytical Chemistry, Faculty of Chemical and Food Technology, Slovak University of Technology, Radlinského 9, SK-812 37 Bratislava, Slovak Republic; b Institute of Organic Chemistry, Catalysis and Petrochemistry, Faculty of Chemical and Food Technology, Slovak University of Technology, Radlinského 9, SK-812 37 Bratislava, Slovak Republic

## Abstract

In the mol­ecular structure of the title compound, C_14_H_21_NO_5_, the six-membered ring of the indolizine moiety adopts a chair conformation. There are two independent mol­ecules in the asymmetric unit. The oxopyrrolidine ring attached to the indolizine ring system is nearly planar, with mean deviations of 0.018 (3) and 0.010 (3) Å for the two mol­ecules. The absolute configuration of the title compound was assigned from the synthesis.

## Related literature

For indolizine derivatives, see: Gubin *et al.* (1992 ▶); Gupta *et al.* (2003 ▶); Liu *et al.* (2007 ▶); Medda *et al.* (2003 ▶); Molyneux & James (1982 ▶); Nash *et al.* (1988 ▶); Pearson & Guo (2001 ▶); Ruprecht *et al.* (1989 ▶); Smith *et al.* (2007 ▶); Teklu *et al.* (2005 ▶). For ring conformations, see: Cremer & Pople (1975 ▶). For the synthesis, see: Šafář *et al.* (2010 ▶). For related structures, see: Brown & Corbridge (1954 ▶); Pedersen (1967 ▶).
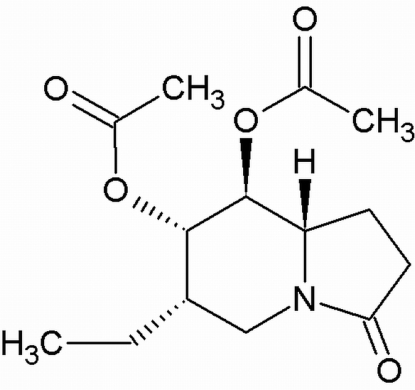


## Experimental

### 

#### Crystal data

C_14_H_21_NO_5_
*M* *_r_* = 283.32
Monoclinic, 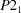

*a* = 11.5157 (2) Å
*b* = 9.8239 (1) Å
*c* = 14.0922 (2) Å
β = 99.035 (2)°
*V* = 1574.46 (4) Å^3^
*Z* = 4
Mo *K*α radiation
μ = 0.09 mm^−1^
*T* = 298 K
0.40 × 0.30 × 0.20 mm

#### Data collection

Oxford Diffraction Gemini R CCD diffractometer
Absorption correction: multi-scan (*CrysAlis PRO*; Oxford Diffraction, 2009 ▶) *T*_min_ = 0.952, *T*_max_ = 0.984
37222 measured reflections
3404 independent reflections
2508 reflections with *I* > 2σ(*I*)
*R*_int_ = 0.028

#### Refinement

*R*[*F* ^2^ > 2σ(*F* ^2^)] = 0.037
*wR*(*F* ^2^) = 0.104
*S* = 1.03
3404 reflections
361 parameters
1 restraint
H-atom parameters constrained
Δρ_max_ = 0.11 e Å^−3^
Δρ_min_ = −0.14 e Å^−3^

### 

Data collection: *CrysAlis PRO* (Oxford Diffraction, 2009 ▶); cell refinement: *CrysAlis PRO*; data reduction: *CrysAlis PRO*; program(s) used to solve structure: *SHELXS97* (Sheldrick, 2008 ▶); program(s) used to refine structure: *SHELXL97* (Sheldrick, 2008 ▶); molecular graphics: *DIAMOND* (Brandenburg, 2001 ▶); software used to prepare material for publication: *SHELXL97*, *PLATON* (Spek, 2009 ▶) and *WinGX* (Farrugia, 1999 ▶).

## Supplementary Material

Crystal structure: contains datablock(s) I, global. DOI: 10.1107/S1600536812005144/bq2336sup1.cif

Structure factors: contains datablock(s) I. DOI: 10.1107/S1600536812005144/bq2336Isup2.hkl

Supplementary material file. DOI: 10.1107/S1600536812005144/bq2336Isup3.cml

Additional supplementary materials:  crystallographic information; 3D view; checkCIF report

## Figures and Tables

**Fig. 1.**
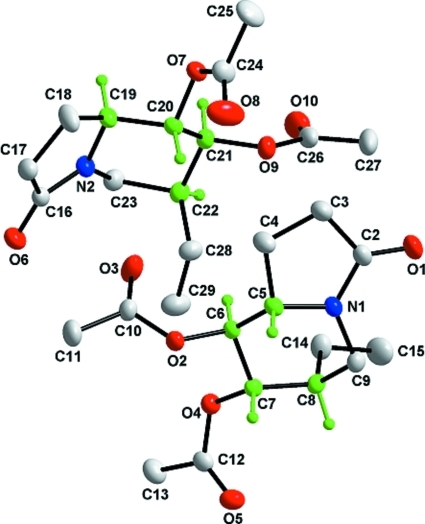
Molecular structure of the title compound showing the atom labeling scheme of the two independent molecules. Displacement ellipsoids are drawn at the 50% probability level (Brandenburg, 2001).

## supplementary crystallographic information

### Comment

Heterocycles are involved in a wide range of biologically important chemical reactions in living organisms, and therefore they form one of the most important and well investigated classes of organic compounds. One group of heterocycles, indolizines, has received much scientific attention during the recent years. Indolizine derivatives have been found to possess a variety of biological activities such as antibacterial, antiinflammatory, antiviral, (Nash *et al.*, 1988; Molyneux & James, 1982; Medda *et al.*, 2003), anti-HIV (Ruprecht *et al.*, 1989), anti-cancer (Liu *et al.*, 2007; Smith *et al.*, 2007), and antitumor (Pearson & Guo, 2001). They have also shown to be calcium entry blockers (Gupta *et al.*, 2003) and potent antioxidants inhibiting lipid peroxidation in vitro (Teklu *et al.*, 2005). As such, indolizines are important synthetic targets in view of developing new pharmaceuticals for the treatment of cardiovascular diseases (Gubin *et al.*, 1992). Based on these facts and in continuation of our interest in developing simple and efficient route for the synthesis of novel indolizine derivatives. We report here the synthesis, molecular and crystal structure of the title compound, (I), which crystallizes in the monoclinic space group P21 with two crystallographic independent molecules in asymmetric unit. The absolute configuration was established by synthesis. The expected stereochemistry of atoms C5, C6, C7 and C8 for molecule A and C19, C20, C21 and C22 for molecule B was confirmed for all as S, see Fig. 1. The central six-membered N-heterocyclic ring is not planar and adopts a chair conformation (Cremer & Pople, 1975). A calculation of least-squares planes shows that this ring is puckered in such a manner that the four atoms C5, C6, C8 and C9 (C19, C20, C22 and C23 for molecule B) are coplanar to within 0.018 (2)Å [0.019 (2)Å], while atoms N1 (N2) and C7 (C21) are displaced from this plane on opposite sides, with out-of-plane displacements of -0.593 (2)Å and 0.659 (2)Å [-0.581 (1)Å and 0.671 (2)Å for molecule B], respectively. In the molecule structure, the oxopyrrolidine ring N1/C2—C5 (N2/C16—C19) attached to the central six-membered ring is nearly planar (mean deviation is 0.018 (3)Å for molecule A and 0.010 (3)Å for molecule B). The dihedral angle between the plane of oxopyrrolidine ring and the plane of the four atoms C5, C6, C8 and C9 (C19, C20, C22 and C23) forming the base of the chair conformation is 51.0 (1)° (54.4 (1)°). The N1 (N2) atom is sp2 hybridized, as evidenced by the sum of the valence angles around it [359.9 (2)° for molecule A and 359.9 (2)° for molecule B]. These data are consistent with conjugative delocalization of the lone-pair electrons on N1 (N2) atom with the adjacent carbonyl C2=O1 (C16=O6) and agree with literature values for simple amides (Brown & Corbridge, 1954; Pedersen, 1967). The bond length of the carbonyl group C2=O1 (C16=O6) is 1.220 (3)Å [1.214 (3)Å], respectively, is somewhat longer than typical carbonyl bonds. This may be due to the fact that atoms O1 and O6 participate in intra- or intermolecular C—H···O contacts. The crystal structure is stabilized by weak intra- and intermolecular C—H···O hydrogen bonds.

### Experimental

The title compound was prepared according to a standard protocol described in literature (Šafář *et al.*, 2010).

### Refinement

All H atoms were positioned with idealized geometry using a riding model with C—H distances in the range 0.93 - 0.98 Å. The *U*_iso_(H) values were set at 1.2 *U*_eq_(C-aromatic) or 1.5 *U*_eq_(*C*-methyl). The absolute configuration could not be reliably determined for this compound using Mo radiation, and has been assigned according to the synthesis.

### Crystal data

**Table:**

C_14_H_21_NO_5_	*F*(000) = 608
*M**_r_* = 283.32	*D*_x_ = 1.195 Mg m^−^^3^
Monoclinic, *P*2_1_	Mo *K*α radiation, λ = 0.71073 Å
Hall symbol: P 2yb	Cell parameters from 37222 reflections
*a* = 11.5157 (2) Å	θ = 4.1–26.4°
*b* = 9.8239 (1) Å	µ = 0.09 mm^−^^1^
*c* = 14.0922 (2) Å	*T* = 298 K
β = 99.035 (2)°	Prism, colourless
*V* = 1574.46 (4) Å^3^	0.40 × 0.30 × 0.20 mm
*Z* = 4	

### Data collection

**Table:**

Oxford Diffraction Gemini R CCD diffractometer	3404 independent reflections
Radiation source: fine-focus sealed tube	2508 reflections with *I* > 2σ(*I*)
Graphite monochromator	*R*_int_ = 0.028
Detector resolution: 10.4340 pixels mm^-1^	θ_max_ = 26.4°, θ_min_ = 4.1°
Rotation method data acquisition using ω and φ scans	*h* = −14→14
Absorption correction: multi-scan (*CrysAlis PRO*; Oxford Diffraction, 2009)	*k* = −12→12
*T*_min_ = 0.952, *T*_max_ = 0.984	*l* = −17→17
37222 measured reflections	

### Refinement

**Table:**

Refinement on *F*^2^	Primary atom site location: structure-invariant direct methods
Least-squares matrix: full	Secondary atom site location: difference Fourier map
*R*[*F*^2^ > 2σ(*F*^2^)] = 0.037	Hydrogen site location: inferred from neighbouring sites
*wR*(*F*^2^) = 0.104	H-atom parameters constrained
*S* = 1.03	*w* = 1/[σ^2^(*F*_o_^2^) + (0.0702*P*)^2^] where *P* = (*F*_o_^2^ + 2*F*_c_^2^)/3
3404 reflections	(Δ/σ)_max_ < 0.001
361 parameters	Δρ_max_ = 0.11 e Å^−^^3^
1 restraint	Δρ_min_ = −0.14 e Å^−^^3^

### Special details

**Table:**

Geometry. All e.s.d.'s (except the e.s.d. in the dihedral angle between two l.s. planes) are estimated using the full covariance matrix. The cell e.s.d.'s are taken into account individually in the estimation of e.s.d.'s in distances, angles and torsion angles; correlations between e.s.d.'s in cell parameters are only used when they are defined by crystal symmetry. An approximate (isotropic) treatment of cell e.s.d.'s is used for estimating e.s.d.'s involving l.s. planes.
Refinement. Refinement of *F*^2^ against ALL reflections. The weighted *R*-factor *wR* and goodness of fit *S* are based on *F*^2^, conventional *R*-factors *R* are based on *F*, with *F* set to zero for negative *F*^2^. The threshold expression of *F*^2^ > σ(*F*^2^) is used only for calculating *R*-factors(gt) *etc*. and is not relevant to the choice of reflections for refinement. *R*-factors based on *F*^2^ are statistically about twice as large as those based on *F*, and *R*- factors based on ALL data will be even larger.

### Fractional atomic coordinates and isotropic or equivalent isotropic displacement parameters (Å^2^)

**Table:**

	*x*	*y*	*z*	*U*_iso_*/*U*_eq_	Occ. (<1)
C2	0.8545 (2)	−0.0286 (3)	0.56842 (19)	0.0737 (7)	
C3	0.7241 (3)	−0.0115 (3)	0.5372 (3)	0.0989 (9)	
H3B	0.6979	−0.0639	0.4794	0.119*	
H3A	0.6813	−0.0420	0.5873	0.119*	
C4	0.7043 (3)	0.1376 (3)	0.5183 (3)	0.0889 (8)	
H4B	0.6572	0.1759	0.5630	0.107*	
H4A	0.6637	0.1521	0.4534	0.107*	
C5	0.8254 (2)	0.2041 (2)	0.53147 (16)	0.0638 (6)	
H5A	0.8418	0.2376	0.4694	0.077*	
C6	0.84460 (18)	0.3181 (2)	0.60532 (14)	0.0556 (5)	
H6A	0.8144	0.2913	0.6638	0.067*	
C7	0.97364 (18)	0.3545 (2)	0.62885 (14)	0.0575 (5)	
H7A	0.9995	0.3922	0.5712	0.069*	
C8	1.05094 (19)	0.2324 (3)	0.66341 (15)	0.0631 (5)	
H8A	1.1331	0.2614	0.6681	0.076*	
C9	1.0288 (2)	0.1237 (3)	0.58517 (16)	0.0683 (6)	
H9B	1.0561	0.1560	0.5275	0.082*	
H9A	1.0722	0.0417	0.6065	0.082*	
C10	0.6967 (2)	0.4880 (3)	0.60542 (19)	0.0747 (7)	
C11	0.6557 (3)	0.6217 (4)	0.5632 (3)	0.1127 (11)	
H11C	0.5929	0.6551	0.5942	0.169*	0.50
H11B	0.6281	0.6109	0.4957	0.169*	0.50
H11A	0.7197	0.6854	0.5724	0.169*	0.50
H11F	0.7009	0.6458	0.5140	0.169*	0.50
H11E	0.6657	0.6900	0.6125	0.169*	0.50
H11D	0.5741	0.6155	0.5359	0.169*	0.50
C12	1.0680 (2)	0.5503 (3)	0.70642 (16)	0.0670 (6)	
C13	1.0580 (3)	0.6588 (3)	0.7779 (2)	0.0993 (9)	
H13C	1.1180	0.7259	0.7753	0.149*	0.50
H13B	1.0673	0.6198	0.8411	0.149*	0.50
H13A	0.9821	0.7011	0.7635	0.149*	0.50
H13F	0.9935	0.6386	0.8113	0.149*	0.50
H13E	1.0443	0.7447	0.7455	0.149*	0.50
H13D	1.1295	0.6634	0.8231	0.149*	0.50
C14	1.0331 (3)	0.1806 (3)	0.76184 (18)	0.0828 (7)	
H14B	1.0357	0.2573	0.8055	0.099*	
H14A	0.9557	0.1399	0.7566	0.099*	
C15	1.1237 (3)	0.0773 (4)	0.8039 (3)	0.1224 (13)	
H15C	1.1077	0.0489	0.8657	0.184*	
H15B	1.2006	0.1174	0.8107	0.184*	
H15A	1.1204	−0.0001	0.7620	0.184*	
C16	0.5132 (2)	0.5197 (3)	0.86454 (19)	0.0705 (6)	
C17	0.4201 (2)	0.5033 (3)	0.7765 (2)	0.0829 (7)	
H17B	0.3542	0.5636	0.7800	0.099*	
H17A	0.4523	0.5238	0.7186	0.099*	
C18	0.3821 (3)	0.3583 (3)	0.7768 (3)	0.1040 (10)	
H18B	0.3932	0.3143	0.7172	0.125*	
H18A	0.2996	0.3527	0.7830	0.125*	
C19	0.4576 (2)	0.2884 (3)	0.86243 (18)	0.0696 (6)	
H19A	0.4068	0.2543	0.9068	0.084*	
C20	0.5353 (2)	0.1748 (2)	0.83702 (15)	0.0602 (5)	
H20A	0.5746	0.2018	0.7831	0.072*	
C21	0.6251 (2)	0.1355 (2)	0.92278 (15)	0.0629 (6)	
H21A	0.5844	0.0979	0.9729	0.076*	
C22	0.69956 (19)	0.2565 (3)	0.96345 (15)	0.0639 (6)	
H22A	0.7486	0.2263	1.0229	0.077*	
C23	0.6164 (2)	0.3658 (3)	0.99042 (16)	0.0716 (6)	
H23B	0.5773	0.3333	1.0422	0.086*	
H23A	0.6606	0.4469	1.0124	0.086*	
C24	0.4582 (3)	0.0020 (3)	0.7247 (2)	0.0877 (8)	
C25	0.3881 (4)	−0.1254 (4)	0.7148 (3)	0.1301 (14)	
H25C	0.3881	−0.1631	0.6519	0.195*	0.50
H25B	0.3087	−0.1058	0.7235	0.195*	0.50
H25A	0.4220	−0.1898	0.7624	0.195*	0.50
H25F	0.3578	−0.1427	0.7733	0.195*	0.50
H25E	0.4371	−0.1999	0.7017	0.195*	0.50
H25D	0.3239	−0.1160	0.6628	0.195*	0.50
C26	0.7488 (2)	−0.0597 (3)	0.9512 (3)	0.0968 (9)	
C27	0.8059 (3)	−0.1700 (4)	0.9024 (4)	0.1403 (16)	
H27C	0.8445	−0.2322	0.9497	0.210*	0.50
H27B	0.8627	−0.1310	0.8673	0.210*	0.50
H27A	0.7473	−0.2178	0.8590	0.210*	0.50
H27F	0.7918	−0.1551	0.8343	0.210*	0.50
H27E	0.7737	−0.2564	0.9167	0.210*	0.50
H27D	0.8890	−0.1695	0.9250	0.210*	0.50
C28	0.7819 (2)	0.3082 (3)	0.89634 (17)	0.0788 (7)	
H28B	0.8224	0.2311	0.8737	0.095*	
H28A	0.7351	0.3503	0.8408	0.095*	
C29	0.8716 (3)	0.4091 (5)	0.9418 (3)	0.1207 (13)	
H29C	0.9133	0.4456	0.8937	0.181*	
H29B	0.9260	0.3645	0.9906	0.181*	
H29A	0.8328	0.4815	0.9701	0.181*	
N1	0.90503 (17)	0.09344 (19)	0.56432 (13)	0.0636 (5)	
N2	0.53009 (18)	0.3980 (2)	0.90770 (15)	0.0708 (5)	
O1	0.9073 (2)	−0.1337 (2)	0.59263 (17)	0.1011 (6)	
O2	0.78379 (13)	0.43758 (17)	0.56408 (11)	0.0666 (4)	
O3	0.6567 (2)	0.4302 (3)	0.66728 (17)	0.1181 (8)	
O4	0.98020 (13)	0.46046 (18)	0.70120 (10)	0.0679 (4)	
O5	1.14461 (16)	0.5425 (2)	0.65819 (12)	0.0856 (5)	
O6	0.56358 (19)	0.6243 (2)	0.89226 (16)	0.0923 (6)	
O7	0.46121 (14)	0.05762 (17)	0.81120 (11)	0.0695 (4)	
O8	0.5052 (3)	0.0493 (4)	0.66446 (17)	0.1501 (12)	
O9	0.69467 (16)	0.0294 (2)	0.88760 (13)	0.0826 (5)	
O10	0.7497 (2)	−0.0500 (3)	1.0360 (2)	0.1259 (9)	

### Atomic displacement parameters (Å^2^)

**Table:**

	*U*^11^	*U*^22^	*U*^33^	*U*^12^	*U*^13^	*U*^23^
C2	0.0872 (17)	0.0531 (15)	0.0852 (15)	0.0041 (14)	0.0269 (13)	0.0059 (12)
C3	0.0889 (19)	0.0666 (18)	0.144 (3)	−0.0067 (16)	0.0265 (18)	0.0090 (17)
C4	0.0780 (17)	0.0680 (18)	0.113 (2)	−0.0098 (15)	−0.0081 (14)	0.0068 (16)
C5	0.0670 (13)	0.0564 (13)	0.0662 (12)	−0.0004 (11)	0.0044 (10)	0.0068 (11)
C6	0.0581 (12)	0.0506 (11)	0.0591 (11)	0.0051 (10)	0.0124 (9)	0.0090 (10)
C7	0.0615 (12)	0.0580 (13)	0.0554 (11)	−0.0024 (11)	0.0163 (9)	−0.0059 (10)
C8	0.0573 (12)	0.0677 (14)	0.0644 (12)	0.0057 (12)	0.0103 (9)	−0.0040 (11)
C9	0.0692 (14)	0.0652 (14)	0.0716 (13)	0.0101 (12)	0.0145 (10)	−0.0050 (11)
C10	0.0681 (14)	0.0686 (16)	0.0875 (16)	0.0122 (13)	0.0127 (13)	−0.0018 (13)
C11	0.104 (2)	0.073 (2)	0.161 (3)	0.0268 (19)	0.022 (2)	0.007 (2)
C12	0.0729 (14)	0.0590 (14)	0.0651 (12)	−0.0023 (13)	−0.0017 (11)	0.0075 (11)
C13	0.115 (2)	0.0734 (19)	0.106 (2)	−0.0096 (17)	0.0070 (17)	−0.0213 (16)
C14	0.0967 (18)	0.0839 (18)	0.0657 (14)	0.0198 (15)	0.0066 (13)	0.0070 (13)
C15	0.118 (3)	0.116 (3)	0.124 (2)	0.016 (2)	−0.0097 (19)	0.041 (2)
C16	0.0691 (14)	0.0539 (15)	0.0925 (16)	0.0012 (12)	0.0249 (12)	−0.0094 (13)
C17	0.0702 (15)	0.0698 (17)	0.1057 (18)	0.0084 (13)	0.0048 (13)	0.0078 (14)
C18	0.101 (2)	0.0642 (17)	0.129 (2)	0.0087 (16)	−0.0360 (18)	−0.0061 (17)
C19	0.0644 (13)	0.0557 (14)	0.0862 (15)	−0.0003 (11)	0.0039 (11)	−0.0064 (12)
C20	0.0673 (13)	0.0538 (13)	0.0607 (11)	−0.0043 (11)	0.0134 (10)	0.0006 (10)
C21	0.0661 (13)	0.0633 (14)	0.0612 (12)	0.0090 (12)	0.0156 (10)	0.0058 (11)
C22	0.0646 (12)	0.0730 (16)	0.0538 (11)	0.0046 (12)	0.0084 (9)	−0.0008 (11)
C23	0.0730 (14)	0.0779 (16)	0.0642 (13)	−0.0014 (13)	0.0121 (11)	−0.0122 (12)
C24	0.105 (2)	0.0780 (19)	0.0827 (17)	−0.0088 (16)	0.0217 (15)	−0.0191 (14)
C25	0.171 (4)	0.081 (2)	0.136 (3)	−0.035 (2)	0.017 (3)	−0.043 (2)
C26	0.0648 (16)	0.0689 (19)	0.154 (3)	0.0076 (15)	0.0091 (18)	0.018 (2)
C27	0.090 (2)	0.078 (2)	0.250 (5)	0.0224 (19)	0.018 (3)	−0.022 (3)
C28	0.0731 (15)	0.0922 (19)	0.0735 (14)	−0.0126 (15)	0.0187 (11)	−0.0117 (14)
C29	0.104 (2)	0.137 (3)	0.126 (2)	−0.038 (2)	0.0343 (19)	−0.032 (3)
N1	0.0686 (11)	0.0508 (11)	0.0703 (10)	0.0038 (9)	0.0073 (9)	0.0014 (8)
N2	0.0679 (12)	0.0571 (12)	0.0838 (13)	0.0019 (10)	0.0007 (10)	−0.0106 (10)
O1	0.1129 (15)	0.0586 (11)	0.1369 (17)	0.0145 (12)	0.0351 (13)	0.0165 (12)
O2	0.0688 (9)	0.0574 (9)	0.0749 (9)	0.0083 (8)	0.0157 (7)	0.0120 (8)
O3	0.1130 (15)	0.124 (2)	0.1310 (17)	0.0431 (15)	0.0634 (14)	0.0319 (16)
O4	0.0733 (9)	0.0625 (10)	0.0704 (8)	−0.0020 (9)	0.0185 (7)	−0.0114 (8)
O5	0.0793 (10)	0.0915 (13)	0.0853 (10)	−0.0186 (10)	0.0109 (9)	0.0003 (10)
O6	0.0981 (13)	0.0630 (12)	0.1168 (14)	−0.0122 (11)	0.0203 (11)	−0.0161 (11)
O7	0.0800 (10)	0.0554 (9)	0.0749 (9)	−0.0086 (8)	0.0177 (7)	−0.0080 (8)
O8	0.203 (3)	0.169 (3)	0.0902 (13)	−0.078 (3)	0.0596 (16)	−0.0421 (18)
O9	0.0816 (11)	0.0694 (11)	0.0993 (11)	0.0176 (10)	0.0223 (9)	−0.0020 (10)
O10	0.1194 (18)	0.112 (2)	0.1410 (19)	0.0225 (16)	0.0056 (15)	0.0507 (19)

### Geometric parameters (Å, º)

**Table:**

C2—O1	1.220 (3)	C16—O6	1.214 (3)
C2—N1	1.338 (3)	C16—N2	1.342 (4)
C2—C3	1.506 (4)	C16—C17	1.515 (4)
C3—C4	1.500 (5)	C17—C18	1.490 (4)
C3—H3B	0.9700	C17—H17B	0.9700
C3—H3A	0.9700	C17—H17A	0.9700
C4—C5	1.525 (4)	C18—C19	1.534 (4)
C4—H4B	0.9700	C18—H18B	0.9700
C4—H4A	0.9700	C18—H18A	0.9700
C5—N1	1.451 (3)	C19—N2	1.448 (3)
C5—C6	1.521 (3)	C19—C20	1.509 (3)
C5—H5A	0.9800	C19—H19A	0.9800
C6—O2	1.441 (3)	C20—O7	1.445 (3)
C6—C7	1.514 (3)	C20—C21	1.512 (3)
C6—H6A	0.9800	C20—H20A	0.9800
C7—O4	1.450 (3)	C21—O9	1.449 (3)
C7—C8	1.527 (3)	C21—C22	1.524 (3)
C7—H7A	0.9800	C21—H21A	0.9800
C8—C14	1.521 (4)	C22—C23	1.527 (3)
C8—C9	1.528 (3)	C22—C28	1.527 (3)
C8—H8A	0.9800	C22—H22A	0.9800
C9—N1	1.441 (3)	C23—N2	1.443 (3)
C9—H9B	0.9700	C23—H23B	0.9700
C9—H9A	0.9700	C23—H23A	0.9700
C10—O3	1.192 (3)	C24—O8	1.172 (4)
C10—O2	1.332 (3)	C24—O7	1.331 (3)
C10—C11	1.488 (4)	C24—C25	1.484 (5)
C11—H11C	0.9600	C25—H25C	0.9600
C11—H11B	0.9600	C25—H25B	0.9600
C11—H11A	0.9600	C25—H25A	0.9600
C11—H11F	0.9600	C25—H25F	0.9600
C11—H11E	0.9600	C25—H25E	0.9600
C11—H11D	0.9600	C25—H25D	0.9600
C12—O5	1.197 (3)	C26—O10	1.198 (4)
C12—O4	1.336 (3)	C26—O9	1.335 (4)
C12—C13	1.483 (4)	C26—C27	1.490 (5)
C13—H13C	0.9600	C27—H27C	0.9600
C13—H13B	0.9600	C27—H27B	0.9600
C13—H13A	0.9600	C27—H27A	0.9600
C13—H13F	0.9600	C27—H27F	0.9600
C13—H13E	0.9600	C27—H27E	0.9600
C13—H13D	0.9600	C27—H27D	0.9600
C14—C15	1.508 (5)	C28—C29	1.500 (5)
C14—H14B	0.9700	C28—H28B	0.9700
C14—H14A	0.9700	C28—H28A	0.9700
C15—H15C	0.9600	C29—H29C	0.9600
C15—H15B	0.9600	C29—H29B	0.9600
C15—H15A	0.9600	C29—H29A	0.9600
			
O1—C2—N1	124.7 (2)	C16—C17—H17B	110.6
O1—C2—C3	127.3 (2)	C18—C17—H17A	110.6
N1—C2—C3	108.0 (2)	C16—C17—H17A	110.6
C4—C3—C2	106.1 (2)	H17B—C17—H17A	108.7
C4—C3—H3B	110.5	C17—C18—C19	107.4 (2)
C2—C3—H3B	110.5	C17—C18—H18B	110.2
C4—C3—H3A	110.5	C19—C18—H18B	110.2
C2—C3—H3A	110.5	C17—C18—H18A	110.2
H3B—C3—H3A	108.7	C19—C18—H18A	110.2
C3—C4—C5	106.7 (2)	H18B—C18—H18A	108.5
C3—C4—H4B	110.4	N2—C19—C20	109.42 (18)
C5—C4—H4B	110.4	N2—C19—C18	103.3 (2)
C3—C4—H4A	110.4	C20—C19—C18	115.3 (2)
C5—C4—H4A	110.4	N2—C19—H19A	109.5
H4B—C4—H4A	108.6	C20—C19—H19A	109.5
N1—C5—C6	108.47 (17)	C18—C19—H19A	109.5
N1—C5—C4	103.7 (2)	O7—C20—C19	107.49 (17)
C6—C5—C4	115.5 (2)	O7—C20—C21	107.50 (18)
N1—C5—H5A	109.6	C19—C20—C21	110.83 (18)
C6—C5—H5A	109.6	O7—C20—H20A	110.3
C4—C5—H5A	109.6	C19—C20—H20A	110.3
O2—C6—C7	107.10 (17)	C21—C20—H20A	110.3
O2—C6—C5	108.32 (15)	O9—C21—C20	104.81 (18)
C7—C6—C5	110.86 (17)	O9—C21—C22	112.40 (17)
O2—C6—H6A	110.2	C20—C21—C22	112.06 (19)
C7—C6—H6A	110.2	O9—C21—H21A	109.1
C5—C6—H6A	110.2	C20—C21—H21A	109.1
O4—C7—C6	105.33 (16)	C22—C21—H21A	109.1
O4—C7—C8	112.09 (16)	C21—C22—C23	107.71 (18)
C6—C7—C8	112.78 (18)	C21—C22—C28	113.21 (18)
O4—C7—H7A	108.8	C23—C22—C28	113.2 (2)
C6—C7—H7A	108.8	C21—C22—H22A	107.5
C8—C7—H7A	108.8	C23—C22—H22A	107.5
C14—C8—C7	113.47 (19)	C28—C22—H22A	107.5
C14—C8—C9	113.1 (2)	N2—C23—C22	109.41 (18)
C7—C8—C9	107.24 (16)	N2—C23—H23B	109.8
C14—C8—H8A	107.6	C22—C23—H23B	109.8
C7—C8—H8A	107.6	N2—C23—H23A	109.8
C9—C8—H8A	107.6	C22—C23—H23A	109.8
N1—C9—C8	109.81 (18)	H23B—C23—H23A	108.2
N1—C9—H9B	109.7	O8—C24—O7	123.5 (3)
C8—C9—H9B	109.7	O8—C24—C25	124.9 (3)
N1—C9—H9A	109.7	O7—C24—C25	111.6 (3)
C8—C9—H9A	109.7	C24—C25—H25C	109.5
H9B—C9—H9A	108.2	C24—C25—H25B	109.5
O3—C10—O2	123.1 (2)	H25C—C25—H25B	109.5
O3—C10—C11	125.4 (3)	C24—C25—H25A	109.5
O2—C10—C11	111.5 (3)	H25C—C25—H25A	109.5
C10—C11—H11C	109.5	H25B—C25—H25A	109.5
C10—C11—H11B	109.5	C24—C25—H25F	109.5
H11C—C11—H11B	109.5	H25C—C25—H25F	141.1
C10—C11—H11A	109.5	H25B—C25—H25F	56.3
H11C—C11—H11A	109.5	H25A—C25—H25F	56.3
H11B—C11—H11A	109.5	C24—C25—H25E	109.5
C10—C11—H11F	109.5	H25C—C25—H25E	56.3
H11C—C11—H11F	141.1	H25B—C25—H25E	141.1
H11B—C11—H11F	56.3	H25A—C25—H25E	56.3
H11A—C11—H11F	56.3	H25F—C25—H25E	109.5
C10—C11—H11E	109.5	C24—C25—H25D	109.5
H11C—C11—H11E	56.3	H25C—C25—H25D	56.3
H11B—C11—H11E	141.1	H25B—C25—H25D	56.3
H11A—C11—H11E	56.3	H25A—C25—H25D	141.1
H11F—C11—H11E	109.5	H25F—C25—H25D	109.5
C10—C11—H11D	109.5	H25E—C25—H25D	109.5
H11C—C11—H11D	56.3	O10—C26—O9	123.2 (3)
H11B—C11—H11D	56.3	O10—C26—C27	125.6 (4)
H11A—C11—H11D	141.1	O9—C26—C27	111.1 (4)
H11F—C11—H11D	109.5	C26—C27—H27C	109.5
H11E—C11—H11D	109.5	C26—C27—H27B	109.5
O5—C12—O4	123.0 (2)	H27C—C27—H27B	109.5
O5—C12—C13	125.1 (3)	C26—C27—H27A	109.5
O4—C12—C13	111.9 (2)	H27C—C27—H27A	109.5
C12—C13—H13C	109.5	H27B—C27—H27A	109.5
C12—C13—H13B	109.5	C26—C27—H27F	109.5
H13C—C13—H13B	109.5	H27C—C27—H27F	141.1
C12—C13—H13A	109.5	H27B—C27—H27F	56.3
H13C—C13—H13A	109.5	H27A—C27—H27F	56.3
H13B—C13—H13A	109.5	C26—C27—H27E	109.5
C12—C13—H13F	109.5	H27C—C27—H27E	56.3
H13C—C13—H13F	141.1	H27B—C27—H27E	141.1
H13B—C13—H13F	56.3	H27A—C27—H27E	56.3
H13A—C13—H13F	56.3	H27F—C27—H27E	109.5
C12—C13—H13E	109.5	C26—C27—H27D	109.5
H13C—C13—H13E	56.3	H27C—C27—H27D	56.3
H13B—C13—H13E	141.1	H27B—C27—H27D	56.3
H13A—C13—H13E	56.3	H27A—C27—H27D	141.1
H13F—C13—H13E	109.5	H27F—C27—H27D	109.5
C12—C13—H13D	109.5	H27E—C27—H27D	109.5
H13C—C13—H13D	56.3	C29—C28—C22	114.1 (2)
H13B—C13—H13D	56.3	C29—C28—H28B	108.7
H13A—C13—H13D	141.1	C22—C28—H28B	108.7
H13F—C13—H13D	109.5	C29—C28—H28A	108.7
H13E—C13—H13D	109.5	C22—C28—H28A	108.7
C15—C14—C8	113.6 (3)	H28B—C28—H28A	107.6
C15—C14—H14B	108.9	C28—C29—H29C	109.5
C8—C14—H14B	108.9	C28—C29—H29B	109.5
C15—C14—H14A	108.9	H29C—C29—H29B	109.5
C8—C14—H14A	108.9	C28—C29—H29A	109.5
H14B—C14—H14A	107.7	H29C—C29—H29A	109.5
C14—C15—H15C	109.5	H29B—C29—H29A	109.5
C14—C15—H15B	109.5	C2—N1—C9	126.8 (2)
H15C—C15—H15B	109.5	C2—N1—C5	115.3 (2)
C14—C15—H15A	109.5	C9—N1—C5	117.79 (19)
H15C—C15—H15A	109.5	C16—N2—C23	126.4 (2)
H15B—C15—H15A	109.5	C16—N2—C19	115.5 (2)
O6—C16—N2	125.5 (3)	C23—N2—C19	118.0 (2)
O6—C16—C17	126.5 (3)	C10—O2—C6	118.56 (18)
N2—C16—C17	108.0 (2)	C12—O4—C7	117.92 (17)
C18—C17—C16	105.7 (2)	C24—O7—C20	119.2 (2)
C18—C17—H17B	110.6	C26—O9—C21	117.9 (2)
			
O1—C2—C3—C4	178.3 (3)	C28—C22—C23—N2	70.5 (3)
N1—C2—C3—C4	−2.4 (4)	C21—C22—C28—C29	−169.1 (3)
C2—C3—C4—C5	4.3 (4)	C23—C22—C28—C29	67.9 (3)
C3—C4—C5—N1	−4.5 (3)	O1—C2—N1—C9	1.4 (4)
C3—C4—C5—C6	−123.1 (3)	C3—C2—N1—C9	−178.0 (2)
N1—C5—C6—O2	168.45 (16)	O1—C2—N1—C5	178.7 (2)
C4—C5—C6—O2	−75.6 (2)	C3—C2—N1—C5	−0.7 (3)
N1—C5—C6—C7	51.2 (2)	C8—C9—N1—C2	−123.6 (3)
C4—C5—C6—C7	167.1 (2)	C8—C9—N1—C5	59.2 (3)
O2—C6—C7—O4	63.58 (19)	C6—C5—N1—C2	126.7 (2)
C5—C6—C7—O4	−178.43 (16)	C4—C5—N1—C2	3.3 (3)
O2—C6—C7—C8	−173.87 (15)	C6—C5—N1—C9	−55.8 (2)
C5—C6—C7—C8	−55.9 (2)	C4—C5—N1—C9	−179.1 (2)
O4—C7—C8—C14	49.6 (3)	O6—C16—N2—C23	−4.8 (4)
C6—C7—C8—C14	−69.1 (3)	C17—C16—N2—C23	175.7 (2)
O4—C7—C8—C9	175.27 (17)	O6—C16—N2—C19	178.6 (2)
C6—C7—C8—C9	56.6 (2)	C17—C16—N2—C19	−0.9 (3)
C14—C8—C9—N1	70.6 (3)	C22—C23—N2—C16	−119.1 (3)
C7—C8—C9—N1	−55.3 (2)	C22—C23—N2—C19	57.5 (3)
C7—C8—C14—C15	−169.6 (3)	C20—C19—N2—C16	122.6 (2)
C9—C8—C14—C15	67.9 (3)	C18—C19—N2—C16	−0.8 (3)
O6—C16—C17—C18	−177.2 (3)	C20—C19—N2—C23	−54.4 (3)
N2—C16—C17—C18	2.3 (3)	C18—C19—N2—C23	−177.7 (2)
C16—C17—C18—C19	−2.7 (4)	O3—C10—O2—C6	−9.7 (4)
C17—C18—C19—N2	2.2 (3)	C11—C10—O2—C6	171.5 (2)
C17—C18—C19—C20	−117.2 (3)	C7—C6—O2—C10	−122.9 (2)
N2—C19—C20—O7	168.15 (17)	C5—C6—O2—C10	117.4 (2)
C18—C19—C20—O7	−75.9 (3)	O5—C12—O4—C7	−4.5 (3)
N2—C19—C20—C21	50.9 (3)	C13—C12—O4—C7	175.3 (2)
C18—C19—C20—C21	166.8 (2)	C6—C7—O4—C12	−152.11 (19)
O7—C20—C21—O9	64.3 (2)	C8—C7—O4—C12	84.9 (2)
C19—C20—C21—O9	−178.50 (19)	O8—C24—O7—C20	−6.5 (5)
O7—C20—C21—C22	−173.53 (16)	C25—C24—O7—C20	173.8 (3)
C19—C20—C21—C22	−56.3 (2)	C19—C20—O7—C24	122.2 (2)
O9—C21—C22—C23	175.42 (17)	C21—C20—O7—C24	−118.5 (2)
C20—C21—C22—C23	57.7 (2)	O10—C26—O9—C21	−5.7 (4)
O9—C21—C22—C28	49.4 (3)	C27—C26—O9—C21	174.1 (2)
C20—C21—C22—C28	−68.3 (3)	C20—C21—O9—C26	−154.8 (2)
C21—C22—C23—N2	−55.4 (3)	C22—C21—O9—C26	83.3 (3)
